# The persistence of psychological distress while waiting for pain management

**DOI:** 10.1177/20494637251377761

**Published:** 2025-09-12

**Authors:** Lydia V. Tidmarsh, Richard Harrison, Harriet Wilkinson, Megan Harrington, Deepak Ravindran, Sally Norwood, Katherine A. Finlay

**Affiliations:** 1 School of Psychology and Clinical Language Sciences, 6816 University of Reading, UK; 2 Centre for Integrative Neuroscience and Neurodynamics, 6816 University of Reading, UK; 3 156748 Royal Berkshire Hospital, Pembroke Surgery, Reading, UK

**Keywords:** chronic pain, psychology, pain management, waiting, quantitative, longitudinal

## Abstract

**Objectives:**

Waiting lists for pain management services globally are extensive, exacerbating the burden of chronic pain for patients and service providers. This study aimed to examine the psychological profiles of people living with chronic pain (PLwCP) during long treatment delay and use appropriate inferential analyses of waitlist data to identify potential demographic characteristics presenting at-risk subgroups.

**Method:**

A longitudinal survey design tracked measures of psychological wellbeing (pain self-efficacy, depression, anxiety and pain catastrophizing) in PLwCP (*N* = 211, Males = 50, Females = 161) on the waitlist for pain management, in a major regional NHS hospital in the Southeast of the UK. Measures were collected at baseline, three-months and six-months of waiting.

**Results:**

Regression and ANOVA models revealed that clinically significant levels of depression, anxiety, pain catastrophizing and pain self-efficacy remained high throughout the waiting period, indicating sustained psychological distress. While pain self-efficacy significantly increased over time and though the effect size was small, levels were in the clinically severe range throughout the wait-time, thus requiring intervention. Older and younger adults showed different phenotypical patterns of psychosocial wellbeing whilst waiting.

**Conclusions:**

These findings demonstrate that clinical levels of psychological distress are persistent and entrenched throughout the waitlist for pain management. PLwCP remain an at-risk population in significant need of earlier support. Prehabilitation offers a prospective framework through which early intervention can be achieved. Subgroups identified as greater risk are younger individuals and those with worse depression, anxiety, pain catastrophizing and/or pain self-efficacy upon referral. These factors present stratification targets and direction of where prehabilitation is most urgently required. These findings have clear implications to improve pain practice.

## Introduction

Chronic pain is a global public health burden, estimated to cost over $40.4 billion in Canada,^1^ and $560 to $635 billion in the US.^2^ Chronic pain impacts upon 20% of the population worldwide,^3^ and people living with chronic pain (PLwCP) typically experience high levels of comorbid depression and anxiety,^4^ increasing healthcare strains. While medically acceptable wait-times for chronic pain management are one-month for urgent, and two-months for semi-urgent cases,^5^ globally, waitlists currently range from eight-months to two-years,^6,7^ drastically exceeding recommendations. Critically, long waiting times are associated with greater healthcare utilisation,^8–11^ further increasing economic and service costs.

Compounding the economic impacts, long waitlists for pain management services have detrimental health outcomes on PLwCP; higher levels of pain intensity depression, anxiety, unemployment, substance abuse, financial burden, suicidal ideation and attempts are all associated with extensive waiting.^12,6,13,14^ Reduced quality of life, pain acceptance and trust in services are also observed during long treatment delays.^15,6,16^ Pre-surgically, longer waitlists are associated with worse post-surgical outcomes including pain intensity and interference, quality of life, and psychological and physical function.^17–20^ Such significant clinical differences are observed in as little as eight-weeks of waiting.^21^ However, for outpatient pain management involving a multidisciplinary approach of medical and psychological approaches, evidence is mixed^15^; in a systematic review assessing waiting for multidisciplinary/psychological intervention, eight (of 18) studies observed psychological decline, seven displayed no significant change and three showed improvement.^15^ Many studies included in the systematic review^15^ were community-based populations randomised to active treatment or waitlist control, rather than tracking patients awaiting pain management services. The contextual experience of patients from a pure community sample, and those participating in clinical research trials is likely to vary. Within the latter, participants are ethically entitled to a full explicit description of the treatment protocol and timeline. Whereas those referred clinically in the general population are not ubiquitously furnished with these details, which can facilitate uncertainty and demoralisation.^11,22^ It is plausible that this could impact the psychological wellbeing of PLwCP while waiting for treatment. A study tracking patients awaiting treatment at a tertiary pain clinic in Australia, found stable measures of pain intensity, distress and pain acceptance up to six-months, and decline thereafter up to 2.5 years of waiting.^16^ However, patients were only tracked after their triage appointment and patients considered as urgent and triaged to treatment before six-months were excluded.^16^ Thus, further exploration is needed to determine the real-world impact of long treatment delay for PLwCP as a whole population, tracking from the point of entry to multidisciplinary pain management services.

It is likely that certain subgroups waiting for pain management support may be *more* susceptible to psychological decline. Sex and age differences in pain are widely established; females are more likely to suffer from chronic pain and pain-related psychological distress compared to men.^23–25^ Older adults (>65) display higher pain self-efficacy (PSE), pain acceptance and lower pain catastrophizing (PC) compared to middle-aged (40–64 years) and younger adults (18–39 years).^26^ Further, critical psychosocial factors including PSE, depression, anxiety and PC increase risk and intensity of chronic pain, diminish quality of life and increase disability.^27–29,30^ Examining whether these factors influence psychological wellbeing throughout the waitlist would identify ‘at risk’ subgroups in need of earlier clinical intervention.

This study aimed to quantitatively assess the scope of outpatient pain management waitlists and associated psychological impacts. Specifically, to address the gaps in literature, this study aimed to examine the point of *entry* to pain management services until triage. In this study, ‘*waiting for pain management*’ refers to the period during which patients are awaiting access to a comprehensive, multidisciplinary pain management service, including medical, psychological and physical rehabilitation strategies. By longitudinally tracking psychological wellbeing (PSE, depression, anxiety and PC) in PLwCP on the waitlist for pain management, this research aimed to examine the psychological profiles and dynamics of PLwCP during long treatment delay and identify potential demographic characteristics presenting ‘at risk’ subgroups.

## Method

### Design

This study used a longitudinal survey design, across three time-points (baseline [entry to the pain management unit], three- and six-months).

### Participants

**Figure 1.**
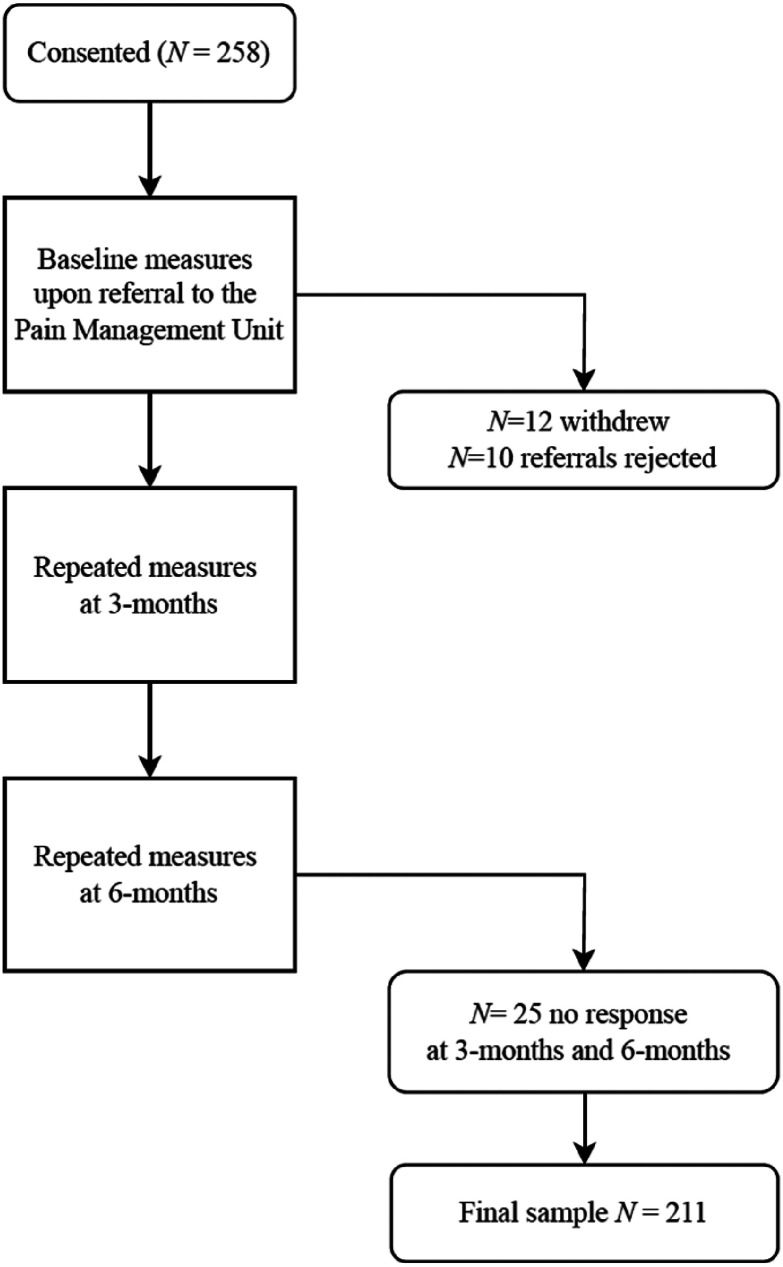
Participant recruitment flow chart.

PLwCP on the waitlist for pain management at Royal Berkshire Hospital NHS Trust were invited to take part in the study upon entry to the multidisciplinary service. No NHS treatment for pain management was received while on the waitlist. A total of 258 participants consented to take part, 12 withdrew via email, 25 decided not to continue with longitudinal assessments after baseline and 10 referrals were rejected by the Pain Management Unit before triage. Therefore, the final sample was *N* = 211, aged between 18 and 89 years (M = 51.5, SD = 15.4). The inclusion criteria for participation were participants aged 18 years or above, on the formal clinical waiting list of the Pain Management Unit and a diagnosis of chronic pain. Overall, our participants represent a clinical sample of chronic pain patients, seeking treatment, after their referral to a Pain Management Unit, at a large regional hospital. Participant demographics are presented in Table 1 and the participant recruitment flow chart is presented in Figure 1.

**Table 1.** Participant characteristics.

	*N*	Percentage
Total sample	211	100%
Gender		
Male	50	23.7%
Female	161	76.3%
Age		
Mean age (years)	51.5	
Age range (years)	18–89	
Age SD	15.4	
Chronic pain duration		
Mean chronic pain duration (years)	9.2	
Range chronic pain duration (years)	1–55	
Chronic pain duration SD	10.3	
Employment status		
Employed	91	43.1%
Unemployed	119	56.4%
Unable to work due to pain	77	33.6% (Total sample) 64.7% (Unemployed)
Missing	1	0.5%
Ethnicity		
White British	138	65.5%
White Irish	2	0.9%
White - any other white background	6	2.8%
Unknown	16	7.6%
Other - not stated	30	14.2%
Other - any other ethnic group	9	4.3%
Mixed - any other background	1	0.5%
Black or Black British - Caribbean	2	0.9%
Black - any other black background	1	0.5%
Asian or Asian British - Indian	4	1.9%
Asian - any other Asian background	2	0.9%

### Materials

Four questionnaires were used alongside a pain history assessment, as follows:

Pain history detailing length of pain condition, location of pain, perceived cause of pain, current employment status and comorbid medical conditions.

Pain Self-Efficacy Questionnaire (PSE-Q)^31^: a 10-item measure of self-belief in ability to manage one’s own pain, showing excellent validity, reliability, and responsiveness in chronic pain samples.^32^ Items are measured on a seven-point Likert scale, the total score ranging from 0 to 60, with higher scores indicating higher pain self-efficacy.

The Patient Health Questionnaire-9 (PHQ-9)^33^: a nine-item brief measure of clinical depression and a widely used, reliable and valid measure of depression severity in outpatient clinical populations.^34^ Items are measured on a four-point Likert scale, total score ranging from 0 to 27, with higher scores indicating higher depression levels.

The Generalised Anxiety Disorder-7 (GAD-7)^35^: a seven-item questionnaire developed to identify probable cases of GAD and measure the severity of GAD symptoms, showing test–retest reliability and validity.^36^ Items are measured on a four-point Likert scale, total score ranging from 0–21, with higher scores indicating higher anxiety levels.

Pain Catastrophizing Scale (PCS)^37^: a 13-item measure of negative pain catastrophizing, demonstrating good internal and test–retest reliability.^38^ Items are measured on a five-point Likert scale, with total score ranging from 0 to 52, with higher scores indicating higher pain catastrophizing levels.

### Procedure

Upon referral to the pain management service, where patients enter the waiting list, people were invited to participate in the study via email. Information sheets were provided, and participants were informed their involvement in the study would have no influence on their clinical care/care pathway, and of their right to withdraw at any point without reason. All measures included in the research are standardised assessment measures collected by the pain management unit for all clients upon entry to the service. Following provision of written informed consent, the measures were repeated at three and six-month intervals while waiting. This study adhered to the code of ethical conduct of the Declaration of Helsinki^39^ and received ethical approval from the Univeristy of Reading ethics committee (UREC:22/06) and the United Kingdom Health Research Authority (HRA:22/NW/0059, IRAS 302397).

### Analysis

#### Data management: Missing data

Missing values analysis showed that 6.11% of the data was missing. Little’s test confirmed the missing data was MCAR (χ^2^(84) = 15.4, *p* = 1.000). Therefore, multiple imputation (MI) was performed using PMM, imputing missing values for the continuous variables. All predictive variables were inputted within the imputation model. Descriptive and inferential analyses were performed via SPSS and where pooling was not available in SPSS, statistics were pooled in R 4.4.2 using Rubin’s rules.^40^

#### Statistical analyses

ANOVA and regression models were used for the statistical analyses. To investigate the presence of psychological change over time, a one-way repeated measures ANOVA was conducted to determine whether there was a statistically significant difference in psychometric scores from baseline to three-months and/or six-months of waiting. To examine potential sex differences in psychometrics over time, a mixed (between-within subjects) ANOVA was used. The within subject variable of Time (baseline, three-months and six-months) assessed longitudinal variation, with sex used to interrogate potential variation in psychometric scores between male and female patients. To investigate potential predictive markers of psychological wellbeing over time, linear regressions were conducted, and due to the large variability in age, this was used to investigate this as a potential individual differences variable of interest. A series of linear regressions were performed to establish whether age was a predictor of each psychometric variable (PSE, depression, anxiety and PCS) at baseline, three-months and six-months. To interrogate individual differences and evaluate whether features of psychology can be predictive of risk-factors to poor psychological wellbeing while waiting, thus classifying potential phenotypes, a series of linear regressions were conducted to establish whether baseline levels of each psychometric variable (PSE, depression, anxiety and PCS) predict levels at three-months and six-months.

#### Assumptions testing

Assumptions testing was completed for all tests. For ANOVAs, boxplots were visually inspected, determining no extreme outliers and Mauchly’s Test of Sphericity indicated that the assumption of sphericity was met. Levene’s Test indicated no significant violations of the homogeneity of variance assumption for any dependent variable at any time point. Normality was violated for all dependent variables as indicated by Shapiro–Wilk’s test <.001 and confirmed by the histogram and Q-Q plots. As all other assumptions were met, and the sample size *N* = 211, the Central Limit theorem ensures robust and reliable ANOVA results despite non-normality.^41,42^ Bootstrapping was not performed as evidence shows MI without bootstrap is robust against skewness.^43^ However, for sensitivity testing, a non-parametric alternative was also performed. A Friedman’s Test was conducted to compare pain self-efficacy scores across three time-points (baseline, three-months, and six-months).

For regression models, visual inspection of the scatterplot indicated a linear relationship between the variables. There was homoscedasticity as assessed by visual inspection of a plot of standardised residuals versus standardised predicted values. Residuals were normally distributed as assessed by visual inspection of the normal probability plots. The assumption of independence of residuals was met, as indicated by Durbin-Watson statistic for each regression model.

## Results

### Pain management waiting list profile

Mean waiting times between referral to the Pain Management Unit to triage were 166 days (range 19 – 436, SD = 90). For the first treatment appointment, mean waiting times were 354 days from referral (range 123 – 637, SD 126) and 193 from triage (range 3 – 510, SD 124). Waiting times for triage and treatment are presented in Table 2.

**Table 2.** Waiting times for triage and treatment.

Triage Waiting Time	Days
Mean time spent waiting for triage	166
Range time spent waiting for triage	19–436
SD time spent waiting for triage	90
Treatment Waiting Time	
Mean time spent waiting from referral to treatment	354
Range time spent waiting from referral to treatment	123–637
SD time spent waiting from referral to treatment	126
Mean time spent waiting from triage to treatment	193
Range time spent waiting from triage to treatment	3–510
SD time spent waiting from triage to treatment	124

#### Demographic characteristics within the waiting list

When examining sex differences in psychometrics over time, a one-way mixed (within-between subjects) ANOVA revealed there was no significant interaction between Time and Sex for PSE (*F*(1, 208) = 1.23, *p* = .32, η^2^ = 0.01), depression (*F*(1, 208) = 1.13, *p* = .35, η^2^ = 0.01), anxiety (*F*(1, 208) = 1.14, *p* = .37, η^2^ = 0.01), or PC (*F*(1, 208) = 1.51, *p* = .23, η^2^ = 0.01). Therefore, sex did not impact upon psychological outcomes when waiting for treatment.

Inspecting individual differences in psychometrics further, a linear regression established no significant association between age and PSE levels at baseline, (*p* = .229), three-months (*p* = .105), or six-months (*p* = .059). Age significantly predicted depression levels at baseline, *F*(1, 209) = 15.14, *p* = .001, explaining 6.8% of the variance (R^2^ = 0.068, Adjusted R^2^ = 0.002), three-months *F*(1, 209) = 12.07, *p* = .001, explaining 5.5% of the variance (R^2^ = 0.055, Adjusted R^2^ = 0.05) and six-months *F*(1, 209) = 4.39, *p* = .006, explaining 3.8% of the variance (R^2^ = 0.038, Adjusted R^2^ = 0.033). While significant, the variance is small. The analysis showed that younger age was positively associated with higher depression scores at baseline (B = −0.12, SE = 0.03, β = −0.26, t(1, 209) = −3.89, *p* < .001), three-months (B = −0.11, SE = 0.03, β = −0.23, t(1, 209) = −3.39, *p* < .001) and six-months (B = −.091, SE = 0.03, β = −0.19, t(1, 209) = −2.80, *p* = .005). Thus, suggesting younger PLwCP report higher levels of depression throughout the waiting time, although age explains only a small proportion of the variability in depression levels.

Age significantly predicts anxiety levels at baseline, *F*(1, 209) = 17.43, *p* = .001, explaining 7.7% of the variance (R^2^ = 0.077, Adjusted R^2^ = 0.073), three-months *F*(1, 209) = 6.40, *p* = .014, explaining 3.0% of the variance (R^2^ = 0.030, Adjusted R^2^ = 0.025) and six-months *F*(1, 209) = 10.90, *p* = .002, explaining 5.0% of the variance (R^2^ = 0.050, Adjusted R^2^ = 0.045). The analysis showed that younger age was positively associated with higher anxiety scores at baseline (B = −0.12, SE = 0.03, β = −0.28, t(1, 209) = −4.18, *p* < .001), three-months (B = −0.07, SE = 0.03, β = −0.17, t(1, 209) = −2.48, *p* = .013) and six-months (B = −0.09, SE = 0.03, β = −0.22, t(1, 209) = −3.23, *p* < .001), indicating younger PLwCP report higher levels of anxiety throughout the waiting time, albeit age explains only a small variance.

Age significantly predicts PC levels at baseline, *F*(1, 209) = 13.24, *p* = .001, explaining 6% of the variance (R^2^ = 0.060, Adjusted R^2^ = 0.055), but no significant association at three-months (*p* = .056) or six-months (*p* = .055). The analysis showed that younger age was positively associated with higher PC scores at baseline (B = −0.22, SE = 0.06, β = −0.24, t(1, 209) = −3.64, *p* < .001), indicating younger PLwCP demonstrate higher levels of PC at baseline, with small variance, which is no longer significantly impacted throughout the waiting period.

A linear regression established age is not a significant predictor of PSE change scores from baseline to three-months (*p* = .358), baseline to six-months (*p* = .285) or change scores from PSE levels at three-months to six-months (*p* = .512), indicating age does not impact change in PSE while waiting. Age is not a predictor of depression change scores from baseline to three-months (*p* = .621), baseline to six-months (*p* = .198), or from three-months to six-months (*p* = .351), indicating age does not impact change in depression throughout the wait-time. Age is a significant predictor of anxiety change scores from baseline to three-months *F*(1, 209) = 5.41, *p* = .026, explaining 2.5% of the variance (R^2^ = 0.025, Adjusted R^2^ = 0.021), but not from baseline to six-months (*p* = .259), or from three-months to six-months (*p* = .352), suggesting the impact of age is only relevant during the initial months of the waitlist, albeit explaining small variance in anxiety levels. Age is a significant predictor of PC change scores from baseline to three-months, *F*(1, 209) = 4.64, *p* = .035, explaining 2.2% of the variance (R^2^ = 0.022, Adjusted R^2^ = 0.017), and from baseline to six-months *F*(1, 209) = 5.37, *p* = .025, explaining 2.5% of the variance (R^2^ = 0.025, Adjusted R^2^ = 0.020), but not change scores from three-months to six-months (*p* = .710), suggesting age explains a small variance in increased PC scores up to 6 months of waiting, but elevated levels remain stable in the latter months. The analysis showed that older age was positively associated with higher change in anxiety scores from baseline to three-months (B = 0.043, SE = 0.20, β = 0.157, t(1, 209) = 2.22, *p* = .027). Older age was positively associated with higher change in PC scores from baseline to three-months (B = 0.098, SE = 0.046, β = 0.147, t(1, 209) = 2.11, *p* = .035), and higher change in PC scores from baseline to six-months (B = 0.113, SE = 0.050, β = 0.158, t(1, 209) = 2.25, *p* = .024). These results indicate older PLwCP demonstrate greater increases in anxiety in the early months of waiting, and greater increases in PC which remain elevated throughout the waitlist.

#### Psychological wellbeing while waiting

**Figure 2.**
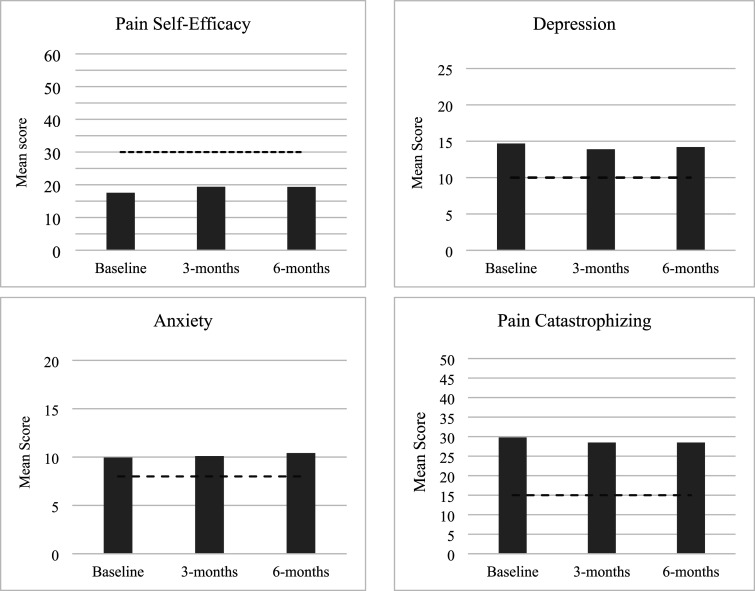
Mean scores of each psychosocial variable at baseline, three-months and six-months. Vertical dotted line showing thresholds of clinical significance, indicating the need for intervention for each variable. *Note.* Depression, anxiety and pain catastrophizing all display higher levels than the cut offs considered as clinically significant, indicating the need for intervention. Pain self-efficacy is much lower than levels considered as clinically significant, requiring intervention.

To investigate the presence of psychological change over time, a one-way ANOVA revealed that PSE scores significantly changed over time *F*(2, 420) = 5.31, *p* = .007, partial η^2^ = 0.03; PSE increased from baseline (Mean = 17.57, SD = 11.94) to three-months (Mean = 19.40, SD = 13.92) and six-months (Mean = 19.35, SD = 13.08), suggesting improvements in PSE while waiting, with a small effect size. There was no statistically significant difference in depression scores (*F*(2, 420) = 2.68, *p* = .077, partial η^2^ = 0.01 ), anxiety scores (*F*(2, 420) = 1.45, *p* = .294, partial η^2^ = 0.01), or PCS scores between time-points (*F*(2, 420) = 2.07, *p* = .130, partial η^2^ = 0.01), indicating that depression, anxiety and PC remained stable over time. For sensitivity testing, Friedman’s test confirmed a significant difference between the PSE time-points (χ^2^(2) = 10.12, *p* = .009). The mean ranks showed PSE scores increased from baseline (1.83) to three-months (2.05) and six-months (2.12). Post-hoc analyses of a Wilcoxon Signed Rank test confirmed a significant difference between PSE levels at baseline (Median = 15) and three-months (Median = 17.13), *z* = −2.76, *p* = .007, and baseline and six-months (Median = 17.38), *z* = −2.63, *p* = .013. There was no significant difference between PSE levels at three-months and six-months, *z* = −0.44, *p* = .696. Thus, a significant increase with a small effect size is only observed from baseline to three-months. Friedman’s Test showed no statistically significant difference in depression scores (χ^2^(2) = 1.02, *p* = .616), anxiety scores (χ^2^(2) = 3.48, *p* = .210), or PCS scores between time-points (χ^2^(2) = 2.94, *p* = .234), confirming that depression, anxiety and PC remained stable over time (Table 3; Figure 2).

**Table 3.** ANOVA results exploring change over time (Baseline, three-months and six-months) for each dependent variable.

Dependent variable	F-value	df1	df2	*p* value	η^2^
PSE	5.31	2	420	0.007*	0.03
Depression	2.68	2	420	0.077	0.01
Anxiety	1.45	2	420	0.294	0.01
PCS	2.07	2	420	0.130	0.01

Due to the presence of likely inter-correlations, a Pearson correlation analysis was conducted to examine the relationship between the four baseline variables: PSE, anxiety, depression and PC. Baseline PSE was significantly negatively correlated with baseline depression (*r* = −0.564, *p* < .001), anxiety (*r* = −0.432, *p* < .001), and PC (*r* = −0.496, *p* < .001). Baseline depression was significantly positively correlated with baseline anxiety (*r* = 0.776, *p* < .001), and PC (*r* = 0.641, p < .001). Baseline anxiety was significantly positively correlated with baseline PC (*r* = 0.729, *p* < .001). Therefore, suggesting that as baseline levels of PSE decrease, levels of depression, anxiety and PC increase. Additionally, PLwCP with higher levels of depression are also significantly likely to have higher levels of anxiety, depression and PC and vice versa. Thus, one element of psychological health has widespread impacts for PLwCP.

To evaluate whether features of psychology can be predictive of risk-factors to poor psychological wellbeing while waiting, a linear regression revealed that baseline levels of PSE significantly predict PSE levels at three-months, *F*(1, 209) = 312.20, *p* < .001, explaining 59.8% of the variance (R^2^ = 0.598, Adjusted R^2^ = 0.597), and at six-months *F*(1, 209) = 170.92, *p* < .001, explaining 44.9% of the variance (R^2^ = 0.449, Adjusted R^2^ = 0.447). Higher baseline PSE scores were positively associated with higher PSE levels at three-months (B = 0.902, SE = 0.052, β = 0.77), t(1, 209) = 17.21, *p* < .001) and at six-months (B = 0.734, SE = 0.06, β = 0.67, t(1, 209) = 12.24, *p* < .001), indicating that people with greater PSE at baseline continue to experience elevated levels across the waiting period.

Baseline depression levels significantly predict depression levels at three-months, *F*(1, 200) = 340.99, *p* < .001, explaining 62% of the variance (R^2^ = 0.620, Adjusted R^2^ = 0.618), and at six-months, *F*(1, 209) = 224.31, *p* < .001, explaining 51.3% of the variance (R^2^ = 0.513, Adjusted R^2^ = 0.511). Higher baseline depression levels were positively associated with higher depression scores at three-months (B = 0.80, SE = 0.45, β = 0.79, t(1, 207) = 17.61, *p* < .001) and at six-months (B = 0.708, SE = 0.05, β = 0.72, t(1, 209) = 13.33, *p* < .001), suggesting that people with higher levels of depression at baseline show continuing levels of depression which remain elevated over time.

Baseline anxiety levels significantly predict anxiety levels at three-months, *F*(1, 209) = 339.34, *p* < .001, explaining 61.8% of the variance (R^2^ = 0.618, Adjusted R^2^ = 0.617), and also at six-months, *F*(1, 206) = 249.93, *p* < .001, explaining 54.4% of the variance (R^2^ = 0.544, Adjusted R^2^ = .542). Higher baseline anxiety scores were positively associated with higher anxiety scores at three-months (B = 0.79, SE = 0.05, β = 0.79, t(1, 209) = 17.52, *p* < .001) and at six-months (B = 0.73, SE = 0.50, β = 0.74, t(1, 209) = 14.48, *p* < .001), showing that people with worse anxiety at baseline sustain higher anxiety levels while waiting.

Pain catastrophizing (PC) levels significantly predict PC levels at three-months, *F*(1, 209) = 239.86, *p* < .001, explaining 53.4% of the variance (R^2^ = 0.534, Adjusted R^2^ = .532), and at six-months, *F*(1, 209) = 149.97, *p* < .001, explaining 41.8% of the variance (R^2^ = 0.418, Adjusted R^2^ = .415) Higher baseline PC scores were positively associated with higher PC scores at three-months (B = 0.74, SE = 0.05, β = 0.73, t(1, 209) = 15.29, *p* < .001) and at six-months (B = 0.57, SE = 0.05, β = 0.65, t(1, 209) = 12.08, *p* < .001), indicating that people with worse PC at baseline have persistently high levels throughout the waiting period (Table 4).

**Table 4.** Regression coefficients for baseline predictors on 3-month and 6-month measures.

	B	SE	β	R^2^	*p* value
IV: PSE Baseline					
PSE three-months	0.902	0.05	0.77	0.598	<.001
PSE six-months	0.734	0.06	0.67	0.449	<.001
IV: Depression baseline					
Depression three-months	0.8	0.45	0.79	0.62	<.001
Depression six-months	0.708	0.05	0.72	0.513	<.001
IV: Anxiety baseline					
Anxiety three-months	0.79	0.05	0.79	0.618	<.001
Anxiety six-months	0.73	0.50	0.74	0.544	<.001
IV: PC baseline					
PC three-months	0.74	0.05	0.73	0.534	<.001
PC six-months	0.57	0.05	0.65	0.418	<.001

#### Potential factors underlying dropout

Preliminary analyses explored if there are differences in baseline scores between the ‘drop-out’ cohort (*N* = 35) and ‘remain’ cohort (*N* = 211), comparing people who remained within the research and those who dropped out. An independent samples t-test revealed that there were no significant differences in the ‘drop-out’ cohort in baseline levels of PSE (M = 14.57, SD = 11.24), compared to the remain cohort (M = 17.66, SD = 11.9), t(246) = 1.43, *p* = .153); no significant difference in baseline depression levels in ‘drop-out’ cohort (M = 15.54, SD = 6.99), compared to the remain cohort (M = 14.61, SD = 7.33), t(246) = −0.70, *p* = .483); no significant difference in baseline anxiety levels in ‘drop-out’ cohort (M = 11.11, SD = 6.79), compared to the remain cohort (M = 9.88, SD = 6.44), t(246) = −1.04, *p* = .299); no significant difference in baseline PC levels in ‘drop-out’ cohort (M = 33.17, SD = 14.21), compared to the remain cohort (M = 29.64, SD = 13.92), t(246) = −1.39, *p* = .166). These results indicate that baseline levels of psychological wellbeing did not influence dropout.

## Discussion

This study aimed to explore the scope of outpatient pain management waitlists, the associated psychological features linked with waiting and the demographic characteristics presented by ‘at risk’ subgroups. Findings show PLwCP were waiting on average, 166 days for triage and 354 days for their first treatment appointment. Overall, there were high levels of persistent psychological distress across the waiting time, with scores falling within the clinically meaningful range (depression >10,^44^ anxiety >8,^35^ PC >15,^37^ and PSE <30^31^). While PSE increased over time, PSE levels were extremely low, with scores falling within the clinically meaningful severe range <30,^31^ indicating psychological intervention is required. PLwCP with higher baseline depression, anxiety and PC continue along an elevated trajectory at three-months and six-months. Those with lower PSE also display consistently lower levels throughout the waiting time. All baseline psychometrics were significantly correlated with each other, suggesting that multi-faceted interventions, which encompass a variety of psychological dimensions, maybe more appropriate than those which target only one. However, this distinction would need formal empirical evaluation prior to application. Baseline levels of psychological wellbeing did not influence withdrawal from research. Sex was not found to influence psychological wellbeing throughout the waitlist. Younger PLwCP present a subgroup demonstrating higher levels of depression and anxiety throughout the waiting time, and higher PC at baseline. However, older PLwCP demonstrate greater increases in anxiety in the early months of waiting, and greater increases in PC which remain elevated throughout the waitlist.

The present study found mean waiting times for triage were 166 days, extending IASP recommendations of 30–60 days for urgent or semi-urgent cases^5^ by 4.4–3.5 months, respectively. From referral to first treatment appointment, PLwCP within this sample were waiting for almost 1 year, on average. These findings coalesce with literature on long treatment delay.^45,11,7^ Policy commitments to improve waitlists and tackle backlogs predominantly focus upon surgical waitlists,^46^ yet these findings confirm outpatient pain waitlists are extensive and medically unacceptable. There is a long time-period that is currently stagnant, which could be activated via earlier intervention.

To fully demonstrate the rationale for earlier intervention for outpatient pain management, the present findings show that PLwCP report clinically significant levels of depression (>10^44^) anxiety (>8^35^) and pain catastrophizing (>15^37^), which remain elevated throughout the currently *unsupported* extensive waiting time. As chronic pain and long treatment delay are associated with a greater risk of suicide ideation and attempts,^47^ this highlights the necessity of earlier intervention. There was no significant deterioration in psychological wellbeing over time, coalescing with mixed patterns evident in pre-existing literature.^15^ As the average waiting time to first treatment appointment in the present study exceeded the six-month tracking period, a decline may be observed beyond six-months.^16^ Tracking was ceased at this point as waiting from the point of *entry* to outpatient pain management services was under-explored, with previous literature focusing on waiting from the point of *triage*.^16^ Our findings demonstrate triage occurred around the six-month point, on average. Thus, taken together, it may be that clinical levels of psychological distress remain stable initially until triage. Being seen and having a care plan put in place at triage may also keep levels relatively stable for six-months,^16^ with decline observed thereafter when PLwCP are waiting for treatment for up to or even beyond one-year.^16^ Chronic pain is typically characterised by pain flare ups, influencing the intensity of psychological comorbidities and scoring on self-report measures.^48^ Therefore, it is likely that as long treatment delay continues, the fluctuating nature of pain will be reflected in reports of psychological wellbeing, and thus potential decline as wait-time continues.^16^ Regardless of deterioration, it is important to recognise that PLwCP present a population who are clinically at risk to poor psychological health which remains throughout the waitlist. Time to be seen for triage is a considerable six-months of unsupported waiting, thus ample time for earlier intervention to be implemented.

While the present findings displayed a significant increase in PSE over time with a small effect size, it is critical to consider the mean scores at each time-point: 17.57 at baseline, 19.4 at three-months and 19.35 at six-months. Scores <30 represent extremely low PSE, considered as clinical levels requiring support.^31^ Therefore, these findings represent a clinical sample in severe need of psychological intervention. Regarding the increase in PSE over time, when exploring themes of psychological distress and self-efficacy, it is important to consider this research-active sample, engaging for six-months, may present phenotype of their own. Evidence shows the act of engaging in clinical research provides an array of benefits including providing a life focus and an improved relationship with their condition.^49^ Therefore, it may be that the group observed are those *most* likely to cope, with higher self-efficacy; there may be a missing group, unidentified here, that are perhaps even more vulnerable. Even so, the extremely low PSE scores evidenced here and high levels of psychological distress, suggest PLwCP are in significant need of support to enhance their perceived capability to manage their pain.

In addition to demonstrating the persistent psychological distress while waiting, our findings identified subgroups of patients evidencing ongoing poor psychosocial wellbeing during long treatment delay; PLwCP who reported higher levels of baseline depression, anxiety and PC, and lower PSE. Collectively, these individuals all demonstrate the same elevated detrimental trajectory in psychological distress while waiting. However, the strong association between chronic pain and depression and anxiety^4^ highlights the relevance of this finding. To protect the psychological health of PLwCP, prehabilitation offers a prospective framework for pre-emptive intervention which could be applied to outpatient pain care, in advance of hospital attendance for multidisciplinary pain management. Prehabilitation, the clinical intervention from the point of diagnosis to eventual treatment,^50^ is successfully utilised pre-operatively, activating patient engagement, improving post-operative outcomes^50–53^ and reducing subsequent healthcare costs^51–53^; (*see*^54^ *for a full review*). The subgroups identified within this study suggest value in stratification to a prehabilitative pathway upon entry to pain management services, based on these psychological factors. Furthermore, these determinants highlight prehabilitative targets of increasing PSE and reducing depression, anxiety and PC, all of which are amenable to change through targeted intervention.^55,56,27,57,58^ This would aim to support and protect patients who are pre-disposed to poor psychological wellbeing during an extensive waiting time that is characterised by uncertainty, anxiety and demoralisation.^6,19,22,59^ The strong baseline correlations are also illuminative; as lower PSE and higher PC are associated with higher depression and anxiety, prehabilitative strategies to enhance self-efficacy and reduce PC are likely to have widespread benefits on psychological wellbeing. Depression and health quality of life are predictive of greater healthcare utilisation and economic cost of care.^60^ Thus, prehabilitation would aim to serve a secondary aim of reducing pain-related healthcare utilisation and economic burdens.

The present findings also suggest older and younger PLwCP showed different phenotypical patterns of psychosocial wellbeing whilst waiting. Younger people are more at-risk of higher depression and anxiety levels at all time-points, and increased PC at baseline. This aligns with evidence displaying a strong association of depression and PC in younger adults, compared to older.^26^ Older chronic pain patients also display lower anxiety, depression and affective distress compared to younger patients.^61^ Our findings may reflect the change in perceptions of pain across age; younger people may be struggling more with the injustice and impact of pain, whereas older people consider pain to be a natural consequence of the ageing process, contributing to pain acceptance.^62^ When exploring change scores from baseline, older age significantly predicts greater increase in PC from baseline to three-months and six-months, and greater increase in anxiety from baseline to three-months. Thus, while younger individuals enter the service with higher anxiety and PC, older age predicts the rate of change in PC and anxiety. However, for anxiety, this effect of age diminishes after the initial period of three-months. This study found no association of age and pain self-efficacy. There is a limited evidence base exploring the relationship between age and pain self-efficacy; however, one study found older adults displayed higher pain self-efficacy compared to younger adults,^26^ contrasting the present findings. Further research is required exploring the cognitive processes involved in pain coping and differences across age. Together, the present results suggest that stratifying prehabilitation pathways based on age may be a protective clinical strategy. Younger PLwCP may benefit from prehabilitation to address consistently higher levels of psychological distress, specifically targeting issues related to age and pain acceptance. While older individuals may require targeted interventions before three-months, to prevent steep increases in anxiety and PC.

Regarding sex differences, this study found no significant association of sex and any psychometric scores at any time point. This contrasts against previous evidence finding women display higher levels of psychological distress, pain-related interference and pain acceptance after waiting for more than six-months, compared to men.^16^ Together, this may suggest that, while sex differences in pain are well established,^23,24^ sex may not be an influential factor in the context of waiting *before* six-months. Additionally, it may be that ill-defined waiting is uniformly distressing for all and that sex differences are therefore minimised. Future research may be required to fully establish if there is a relationship between sex and psychological impacts of waiting, informing the development of a prehabilitation intervention.

### Limitations and future directions

This study examines the impact of waiting on PLwCP on the waitlist for outpatient pain management care. A common challenge when conducting clinical research is engaging the most vulnerable groups, inclusive of those experiencing extremely high levels of psychological distress, for whom additional research-based questionnaires may present as an unwelcome burden.^63^ Future research may offer researcher-assisted or peer-assisted questionnaire completion to support engagement from hard-to-reach groups, building rapport and trust^64^; however it is important to caution this method may carry the risk of introducing demand characteristics or response bias. Due to the nature of longitudinal data, when interpreting the predictive value of the baseline levels of each psychosocial variable at three and six-months, it should be noted that the correlations between these variables are often modest and susceptible to features of autocorrelation, wherein intraindividual longitudinal variables have an inherent relationship with the same variable at an earlier time-point. These features of psychometric, longitudinal research should be taken into consideration when evaluating the strength of the relationships. Standard outcome measures collected within the pain management unit were utilised within this study, with no capacity for additional clinical assessment measure burden. Thus, future research could explore measures of pain-related disability whilst waiting to further explore the impact of long treatment delay on PLwCP. Tracking ceased at six-months as the primary focus of this study was to explore the impact of initial wait-times upon entry to pain management services until triage. Future research could continue longitudinal tracking until treatment receipt, and beyond, to explore patterns of psychosocial wellbeing over a longer time-period of waiting and the impact of waiting on treatment outcomes. However, these findings serve to identify that prehabilitation could have a place in optimising treatment across a varied range of psychological variables. Further empirical work is required to identify specific prehabilitation intervention content and delivery, mapped to the Behaviour Change Wheel (BCW^65^) and Behaviour Change Intervention Ontology (BCIO^66^) for a comprehensive and systematic intervention design. Due to the multi-dimensional nature of the present findings, it is important that a multidisciplinary team is involved in the development of the intervention, building both patient and professional stakeholder involvement into the intervention design.^67^ To ensure prehabilitation does not result in additional waitlists, it is likely an online self-guided intervention would be most appropriate; however, further research is required. To quantify the cost-effectiveness of psychological prehabilitation for outpatient pain management, health economic modelling would be beneficial and insightful. One outstanding question is whether prehabilitation would be optimally compatible with certain treatment options over others (i.e. PMP vs corticosteroid injection). Evidence suggests prehabilitation is effective across a wide array of surgical domains,^50–53^ and maybe versatile across treatments in outpatient pain management also. However, this would need evaluating when implementing prehabilitation at a future stage.

### Conclusions

The current study confirms that waitlists for outpatient pain management are extensive, far exceeding recommendations. Clinical levels of psychological distress are present throughout the entire waiting time, demonstrating that PLwCP remain an at-risk population in significant need of early interventional support. The waitlist presents an opportunity to intervene at an earlier time-point, in advance of pain management service intervention. Prehabilitation offers a successful, prospective framework through which this can be achieved. The findings within this study propose that this model of prehabilitation could be provided proactively in *pre-emptive* readiness while patients are awaiting their upcoming pain management intervention. Further research is required to identify tailored outpatient pain management prehabilitation content and delivery. While distress may not escalate by six-months overall, PLwCP with worse psychological health upon referral and younger patients continue along an elevated trajectory, which persists throughout the wait-time. Prehabilitation may benefit from being tailored by age, targeting issues related to age and pain-related distress and coping. Prehabilitation intervention efforts must be provided to these subgroups with primary importance, to support and protect emotional/psychological health. Upon referral to pain management services, the psychological factors of pain self-efficacy, depression, anxiety and pain catastrophizing provide stratification markers to prehabilitation. These psychological factors also provide insight into critical elements which can be targeted and harnessed within prehabilitation intervention content. As these factors are correlated at baseline, a multi-dimensional approach to prehabilitation is likely to induce widespread psychological benefits. These findings have clear rationale for the need to develop a prehabilitation intervention for outpatient pain management. This would not only protect persistent psychological distress in PLwCP, but it would subsequently reduce excessive healthcare utilisation and economic burdens associated with long treatment delay.
